# The Central Role of Echocardiography in Mavacamten Therapy for Obstructive Hypertrophic Cardiomyopathy: A Comprehensive Review

**DOI:** 10.1155/crp/8857701

**Published:** 2026-08-06

**Authors:** Ali Nizam, Ibrahim Antoun, Liz Hodson, G. André Ng, Harshil Dhutia

**Affiliations:** ^1^ Department of Cardiology, University Hospitals of Leicester NHS Trust, Glenfield Hospital, Leicester, UK, nhs.uk; ^2^ Division of Cardiovascular Sciences, University of Leicester, Leicester, UK, le.ac.uk; ^3^ National Institute for Health Research Leicester Research Biomedical Centre, Leicester, UK; ^4^ Leicester British Heart Foundation Centre of Research Excellence, Glenfield Hospital, Groby Road, Leicester, UK, nhs.uk

**Keywords:** cardiac myosin inhibition, echocardiography, hypertrophic cardiomyopathy, left ventricular outflow tract obstruction, mavacamten

## Abstract

Mavacamten is a cardiac myosin inhibitor used in adults with symptomatic obstructive hypertrophic cardiomyopathy (HCM). Echocardiography is central to selecting suitable patients, confirming left ventricular outflow tract (LVOT) obstruction, excluding inappropriate use when left ventricular ejection fraction (LVEF) is < 55% and guiding dose titration during treatment. This narrative review summarises the practical echocardiographic approach to mavacamten therapy, with emphasis on reproducible LVOT gradient assessment, goal‐directed Valsalva manoeuvres, LVEF monitoring, mitral valve and systolic anterior motion assessment, mitral regurgitation, diastolic function and left atrial size. Current regulatory monitoring requires echocardiographic assessment during early titration and permits 6‐monthly maintenance surveillance only in stable patients meeting defined criteria, including LVEF ≥ 55% and Valsalva LVOT gradient < 30 mmHg, or a decision not to uptitrate. The review also discusses unresolved issues, including exercise echocardiography, global longitudinal strain, atrial fibrillation, reverse remodelling, implementation variability and the broader emergence of cardiac myosin inhibitors such as aficamten. Standardised acquisition and reporting are essential to ensure that treatment decisions reflect true physiological response rather than technical variability.

## 1. Introduction

Obstructive hypertrophic cardiomyopathy (HCM) is characterised by asymmetric or regional left ventricular hypertrophy, dynamic left ventricular outflow tract (LVOT) obstruction, systolic anterior motion (SAM) of the mitral valve, mitral regurgitation (MR), impaired relaxation and increased filling pressures. Transthoracic echocardiography (TTE) is central to diagnosis, risk assessment, therapeutic selection and longitudinal follow‐up [1]. For decades, the treatment of symptomatic obstructive HCM relied on beta‐blockers, nondihydropyridine calcium channel blockers, disopyramide and invasive septal reduction therapy when symptoms persisted despite medical treatment [2].

Mavacamten is a selective allosteric cardiac myosin ATPase inhibitor that reduces the number of actin–myosin cross‐bridges and shifts myosin toward an energy‐sparing super‐relaxed state. In obstructive HCM, this reduces hypercontractility, SAM‐septal contact, LVOT gradients and symptoms [3, 4]. Pivotal clinical trials have shown that mavacamten reduces LVOT gradients and improves symptoms in obstructive HCM. In EXPLORER‐HCM, 6% of the patients receiving mavacamten experienced a transient reduction in left ventricular ejection fraction (LVEF) to < 50%, supporting the need for structured echocardiographic surveillance [3]. In VALOR‐HCM, mavacamten reduced eligibility for septal reduction therapy in patients with severe symptoms and high gradients [5].

A comprehensive baseline TTE is required before initiating treatment to confirm obstructive HCM and to document the dynamic LVOT gradient, wall thickness, SAM, MR severity, LVEF, diastolic function and left atrial size. It is crucial to establish that LVEF is ≥ 55% before treatment because mavacamten has a negative inotropic effect and should not be started below this threshold. Early monitoring is performed at Weeks 4, 8 and 12 after initiation. The 2025 label update permits 6‐monthly monitoring after the titration phase only in stable patients with LVEF ≥ 55% and Valsalva LVOT gradient < 30 mmHg or Valsalva LVOT gradient ≥ 30 mmHg when no uptitration is planned [6, 7].

This review provides a practical and critical synthesis of echocardiography in mavacamten therapy. Its contribution is to integrate regulatory monitoring requirements, HCM imaging guidance, diastolic function assessment, exercise echocardiography, measurement pitfalls and emerging data on cardiac myosin inhibition into a clinically usable framework. We also highlight unresolved questions, including the role of strain imaging, reverse remodelling, atrial fibrillation, broader cardiac myosin inhibitor therapy and real‐world implementation across echocardiography services.

## 2. Baseline Echocardiographic Evaluation Before Mavacamten

### 2.1. LVOT Gradient at Rest and With Provocation

A peak instantaneous LVOT pressure gradient ≥ 50 mmHg at rest or with provocation is generally used as the threshold for considering advanced therapies such as mavacamten or septal reduction. Resting gradients are usually obtained using continuous‐wave Doppler from the apical 5‐chamber or apical long‐axis view, guided by colour Doppler to align with the highest‐velocity LVOT jet. If the resting gradient is < 50 mmHg, provocation should be performed. A goal‐directed Valsalva manoeuvre is preferable to an unguided self‐directed effort because inadequate strain can falsely underestimate latent obstruction. The sonographer should coach the patient to sustain strain for approximately 10–15 s and record during strain and early release. If Valsalva does not elicit a ≥ 50 mmHg gradient and symptoms persist, exercise stress echocardiography is recommended to unmask exertional obstruction [8]. In symptomatic patients without a resting gradient ≥ 50 mmHg, exercise echocardiography is particularly relevant when dyspnoea, chest pain, presyncope or exertional limitation remain unexplained.

### 2.2. Exercise Echocardiography Beyond Gradient Assessment

Exercise echocardiography should not be limited to gradient assessment alone. Comprehensive protocols can evaluate inducible LVOT obstruction, exercise‐induced MR, pulmonary pressures, wall motion abnormalities suggesting ischaemia, chronotropic response and exercise diastolic reserve. Peteiro et al. showed that comprehensive exercise echocardiography is feasible in HCM and adds prognostic information beyond resting imaging. In the International Stress Echo Registry, Ciampi et al. found that stress echocardiography had prognostic value, with ischaemia‐related endpoints showing greater predictive accuracy than haemodynamic endpoints in some analyses [9, 10]. These observations support a broader role for exercise echocardiography when symptoms are disproportionate to resting obstruction or when treatment decisions depend on whether symptoms reflect obstruction, ischaemia, diastolic dysfunction or another mechanism.

### 2.3. Interventricular Septal Thickness and Hypertrophy Pattern

Septal wall thickness should be measured in end‐diastole in standard parasternal long‐axis and short‐axis views, avoiding oblique cuts and trabeculations. A maximum wall thickness ≥ 15 mm in the absence of abnormal loading conditions is diagnostic of HCM in most adults, while 13‐14 mm may support the diagnosis in relatives or genotype‐positive individuals [1]. Because women may have smaller cardiac dimensions and are at risk of delayed recognition when absolute thresholds are applied without clinical context, wall thickness should be interpreted alongside body size, sex, family history, ECG, cardiac magnetic resonance findings and genotype. The distribution of hypertrophy should be described, as asymmetric septal, concentric, apical and midventricular patterns can influence the obstruction level and expected response to therapy. Alternative causes of LV hypertrophy, including hypertension, aortic stenosis, athlete’s heart, storage disease and infiltrative cardiomyopathy, should be excluded when clinically relevant [11].

### 2.4. Mitral Valve Anatomy and SAM

The mitral valve apparatus is integral to obstructive HCM. The baseline echocardiogram should evaluate leaflet morphology, papillary muscle position, chordal abnormalities and congenital anomalies because these features can contribute to SAM and MR. SAM is usually visualised in the parasternal long‐axis view or by M‐mode as anterior mitral leaflet movement toward the septum in systole. Its severity should be documented by the extent and duration of leaflet–septal contact.

### 2.5. MR Severity

Dynamic LVOT obstruction in HCM often causes secondary MR due to SAM and abnormal leaflet, chordal or papillary muscle morphology. This MR is typically eccentric, posteriorly directed and mid‐to‐late systolic. The high‐velocity LVOT jet can distort the colour Doppler field and may be confused with MR. Baseline reports should describe MR severity and mechanism, as intrinsic mitral disease may not fully resolve with gradient reduction [12].

### 2.6. LVEF

A precise measurement of LVEF is critical before starting mavacamten. Mavacamten should not be initiated if LVEF is < 55%, and this threshold should be reported explicitly rather than described using imprecise terms such as preserved, good or normal LVEF. LVOT obstruction in HCM is now understood to be driven primarily by systolic drag forces acting on elongated or anteriorly displaced mitral leaflets, producing SAM and septal contact, rather than by a simple Venturi mechanism. The baseline TTE should measure LVEF using the biplane Simpson method, with contrast when endocardial definition is suboptimal. Three‐dimensional echocardiography may improve reproducibility in hypertrophied ventricles and is useful when available [13].

### 2.7. Diastolic Function and Left Atrial Size

Baseline diastolic assessment helps contextualise symptoms and identify increased LV filling pressures. The 2025 diastolic function recommendations emphasise integrated assessment rather than reliance on a single index, but in HCM, an average *E*/*e* ^′^ > 14 supports increased LV filling pressures when interpreted with mitral inflow, annular *e* ^′^ velocities, tricuspid regurgitation velocity, pulmonary pressures and left atrial volume [14, 15]. Left atrial volume index (LAVI) should be measured at baseline because LA enlargement reflects chronic elevation of filling pressures, MR burden, atrial myopathy or a combination of these factors and is associated with an increased risk of atrial fibrillation. In the EXPLORER‐HCM echocardiographic analysis, mavacamten improved resting *E*/*e* ^′^ and LAVI, supporting the concept that gradient reduction and myosin inhibition may improve markers of diastolic burden and reverse remodelling [16]. The baseline scan should also screen for midventricular obstruction, apical aneurysm, pulmonary hypertension and right heart involvement.

## 3. Standardised Echo Acquisition and Reporting for Reproducibility

Patients starting mavacamten undergo serial echocardiograms to guide dosing and monitor safety. Reproducibility is essential because changes in LVOT gradient or LVEF can alter treatment. Echocardiography laboratories should, therefore, use standardised protocols, sonographer training and consistent reporting templates for myosin inhibitor pathways.

### 3.1. LVOT Gradient Measurement Protocol

Each examination should measure the peak LVOT gradient at rest and with goal‐directed Valsalva manoeuvre unless contraindicated. The patient should be coached to perform a sustained manoeuvre rather than a self‐directed effort, and the quality of the manoeuvre should be documented if suboptimal. The continuous‐wave Doppler cursor should be aligned through the LVOT using colour Doppler, and multiple windows should be used when needed. Heart rate and blood pressure at the time of gradient measurement should be recorded because preload, afterload, hydration, meals, exertion and tachycardia can alter dynamic obstruction. Follow‐up studies should reproduce the same provocation strategy used at baseline unless exercise echocardiography is specifically requested.

### 3.2. LVEF Assessment Method

Because treatment decisions hinge on small changes in LVEF, the same technique should be used at each study. Biplane Simpson measurement from apical 4‐ and 2‐chamber views is appropriate when image quality is adequate. Contrast should be used when endocardial borders are suboptimal, and 3D echocardiography can be used where available for improved reproducibility.

### 3.3. Mitral Valve and SAM Evaluation

At baseline and follow‐up, reports should state whether SAM is present, whether mitral–septal contact is brief or prolonged and whether SAM‐related MR is improving. M‐mode or frame‐by‐frame cine review may help when SAM severity is borderline.

### 3.4. MR Assessment

In obstructive HCM, the apical four‐chamber view often best demonstrates the MR jet. Colour Doppler settings should be consistent across serial studies, with Nyquist limits typically around 50–60 cm/s. Continuous‐wave Doppler of both LVOT flow and MR should be recorded when there is ambiguity because MR is usually higher velocity and mid‐to‐late systolic, whereas LVOT obstruction is typically late‐peaking and dagger‐shaped [17].

### 3.5. Diastolic Function Measurements

Although diastolic indices are not central to immediate dose decisions, consistent evaluation is important for longitudinal assessment. Changes in *E*/*e* ^′^, *e* ^′^ velocities, LAVI, and pulmonary pressures may indicate improvement in filling pressures or reverse remodelling, but these variables remain sensitive to loading conditions, heart rate, MR severity and atrial rhythm.

## 4. Echo‐Guided Dosing and Titration of Mavacamten

One of the unique aspects of mavacamten therapy is the need to individualise dosing based on TTE findings. Echocardiography provides the key metrics, LVOT gradient and LVEF, that inform whether to uptitrate, maintain, downtitrate or interrupt therapy.

In summary, echocardiography‐driven titration of mavacamten is a model of personalised medicine in cardiology. The process requires close collaboration between the echo laboratory and HCM clinicians. Accurate echo data must be interpreted alongside symptoms, rhythm, blood pressure, concomitant medication and drug‐interaction risk. When performed correctly, this strategy maximises gradient reduction while maintaining systolic safety [18, 19].

## 5. Follow‐Up Echo Schedule and REMS Monitoring

After the initial titration period, patients on mavacamten require ongoing echocardiographic surveillance for safety and efficacy. Current monitoring should be described using specific LVEF and Valsalva LVOT gradient thresholds rather than nonspecific terms.

### 5.1. Initial Intensive Monitoring

Early echocardiographic monitoring is performed at Weeks 4, 8 and 12 after treatment initiation because dose adjustments are most likely during this period. Additional scans are required after dose changes.

### 5.2. Maintenance Phase Monitoring

The FDA label update permits required echocardiography every 6 months rather than every 12 weeks only in patients who have reached the maintenance phase at Week 12 or later and meet defined criteria: LVEF ≥ 55% and Valsalva LVOT gradient < 30 mmHg, or Valsalva LVOT gradient ≥ 30 mmHg when no further uptitration is planned [6, 7]. Therefore, monitoring frequency should be linked to explicit LVEF and Valsalva gradient thresholds rather than broad terms such as good EF, preserved EF or minimal gradient.

### 5.3. Monitoring After Dose Adjustments, Interruptions or Drug Interactions

Additional echocardiograms are required when dosing or clinical status changes. If mavacamten is interrupted because of low LVEF or another safety concern, LVEF should be reassessed before restarting and monitored after resumption according to the prescribing pathway. Drug interactions should be described specifically rather than as a generic CYP450 issue: mavacamten is metabolised mainly by CYP2C19 and CYP3A4, and clinically important changes include starting, stopping, or changing strong or moderate CYP2C19 or CYP3A4 inhibitors or inducers, which can alter mavacamten exposure and systolic function risk [6, 7]. New atrial fibrillation, rapid ventricular rates, acute illness or heart failure symptoms should prompt earlier TTE rather than waiting for the next scheduled surveillance scan.

### 5.4. Follow‐Up Duration

General HCM guidance supports periodic cardiac imaging every 1‐2 years even without myosin inhibitor therapy [1, 20]. For patients receiving mavacamten, current regulatory requirements support ongoing echocardiographic surveillance at least every 6 months once maintenance criteria are met. Future evidence may refine this interval, but at present, LVEF and Valsalva LVOT gradient monitoring remain essential safety requirements.

## 6. Recommended Echocardiography Dataset for Mavacamten Follow‐Up

The BSE has published a dedicated guideline for echocardiographic assessment of patients receiving myosin inhibitor therapy, including a minimum dataset [21]. We suggest that each report should include resting LVOT gradient, provoked LVOT gradient with the manoeuvre specified, SAM presence and severity, MR severity and mechanism, LVEF with method, LV dimensions, septal thickness at baseline and interval reassessment, LAVI, diastolic indices, right heart assessment and technical comments.

Global longitudinal strain (GLS) is not mandated for mavacamten dosing but may provide incremental information on subclinical systolic function, myocardial mechanics and reverse remodelling. Serial GLS may be useful when LVEF remains ≥ 50% but symptoms, biomarkers or chamber remodelling raise concern. However, GLS is affected by vendor differences, tracking quality, loading conditions, heart rate and atrial fibrillation; therefore, it should complement rather than replace LVEF and LVOT gradient monitoring.

## 7. Pitfalls in Echocardiographic Assessment and Proposed Solutions

Several common pitfalls may lead to inappropriate treatment decisions if not recognised. These are summarised in Table 1 and include misidentification of MR as LVOT flow, gradient variability due to loading conditions, LVEF measurement error, atrial fibrillation, incorrect localisation of obstruction, blood pressure effects and unrelated pathology.

**TABLE 1:** Common echocardiographic pitfalls in mavacamten therapy and solutions.

Pitfall	Description/Consequence	Solution/Mitigation
The MR jet was mistaken for LVOT flow on Doppler	MR signal is often late systolic and very high velocity and may be mismeasured as the outflow gradient.	Use colour Doppler to distinguish jets. Align the CW cursor with the LVOT and record a separate MR Doppler profile when needed.
Gradient variability due to loading conditions	Hydration status, blood pressure, medications, meals and provocation quality can change the LVOT gradient between studies.	Standardise patient position and provocation. Use goal‐directed Valsalva. Document heart rate and blood pressure.
LVEF measurement error or inconsistency	Poor image quality, foreshortening and inconsistent methodology can produce apparent LVEF changes and inappropriate drug interruption.	Quantify LVEF with biplane Simpson, contrast, or 3D echocardiography. Re‐measure when LVEF approaches key thresholds.
Atrial fibrillation affects interpretation	Irregular cycle length causes beat‐to‐beat variability in filling, gradients and LVEF.	Average multiple beats, optimise rate or rhythm, and repeat assessment after cardioversion when clinically appropriate.
Differentiating the obstruction level	Midventricular obstruction can be mistaken for LVOT obstruction, affecting expected response to mavacamten.	Use colour Doppler and careful imaging to localise obstruction. Document midventricular gradients separately.
Blood pressure influence on gradient and LVEF	Afterload changes can mask or exaggerate obstruction and affect LVEF interpretation.	Record blood pressure during each study. Interpret gradients and LVEF in clinical context.
Changes unrelated to HCM or mavacamten	New wall motion abnormalities, effusion or valve disease may be wrongly attributed to mavacamten.	Correlate with clinical events and use further testing when needed.

## 8. Special Scenarios and Considerations

Borderline baseline LVEF between 50% and 55% represents a high‐risk group. Current labelling discourages initiation below 55% because of the risk of systolic dysfunction. In these patients, LVEF should be confirmed with biplane Simpson, contrast or 3D echocardiography where needed and alternative causes of reduced LVEF should be excluded. If treatment is considered despite borderline LVEF, this should occur only with specialist HCM input, conservative dosing and intensified echocardiographic surveillance. Any decline to < 50% should prompt treatment interruption.

Atrial fibrillation creates both a measurement problem and a therapeutic challenge. Loss of atrial contraction and irregular cycle lengths reduce measurement reliability and can change LVOT gradients while worsening symptoms. Gradient and LVEF assessment require averaging across multiple beats, with rate or rhythm control where possible. New or poorly controlled atrial fibrillation is clinically important during mavacamten therapy because it may precipitate symptoms, alter gradient interpretation and increase the systolic dysfunction risk.

Systemic hypertension alters afterload and can mask or exaggerate dynamic obstruction. Echo reports should document blood pressure at the time of gradient measurement because gradients may increase when afterload is reduced. Other scenarios include paediatric patients, pregnancy, nonobstructive HCM and coexisting coronary disease, where mavacamten use is limited, investigational or contraindicated depending on clinical context [20].

## 9. Broader Role of Echocardiography With Cardiac Myosin Inhibitors

Although this review focuses on mavacamten because it is the currently established agent in routine clinical pathways, echocardiography is likely to remain central to the broader cardiac myosin inhibitor class. Aficamten, a next‐generation cardiac myosin inhibitor, improved exercise capacity in the Phase 3 SEQUOIA‐HCM trial, and its echocardiographic substudy showed reductions in resting and Valsalva LVOT gradients with improvements in LAVI, tissue Doppler *e* ^′^ velocities and *E*/*e* ^′^ over 24 weeks [22, 23].

These data suggest a shared imaging framework for myosin inhibitors: confirm obstructive physiology, quantify gradients reproducibly, monitor LVEF, evaluate SAM and MR and track structural and diastolic remodelling. However, drug‐specific pharmacokinetics, dose algorithms and regulatory monitoring requirements differ, so mavacamten‐specific REMS thresholds should not be applied automatically to aficamten or future agents.

## 10. Unresolved Issues and Implementation Challenges

Several evidence gaps limit a purely protocol‐driven approach. First, the role of GLS in detecting early systolic impairment or reverse remodelling during myosin inhibition remains unresolved. Second, the clinical meaning of reductions in LAVI, *E*/*e* ^′^, wall thickness and biomarkers requires longer follow‐up, especially in patients whose symptoms persist despite gradient reduction.

Third, atrial fibrillation affects LV filling, symptom burden, gradient variability and LVEF interpretation. Fourth, implementation differs across centres because of variation in sonographer training, goal‐directed Valsalva performance, exercise echocardiography availability, access to 3D echocardiography and strain and local HCM‐myosin inhibitor pathways. The proposed dataset should, therefore, be viewed as a practical framework that extends existing guidance by linking echo acquisition directly to treatment decisions, safety thresholds and unresolved clinical scenarios.

## 11. Conclusion

Mavacamten has changed the management of symptomatic obstructive HCM by providing a targeted pharmacological option to reduce SAM‐mediated LVOT obstruction and improve symptoms. Echocardiography is central to safe implementation of therapy, from baseline eligibility assessment to dose titration, safety monitoring and evaluation of treatment response.

A comprehensive baseline echocardiogram confirms true obstructive physiology, documents LVEF ≥ 55%, identifies mitral valve and MR mechanisms and establishes reproducible LVOT gradient and diastolic function measurements. Standardised acquisition, goal‐directed Valsalva, careful Doppler interpretation and consistent LVEF methodology are essential for meaningful longitudinal comparisons.

**FIGURE 1:**
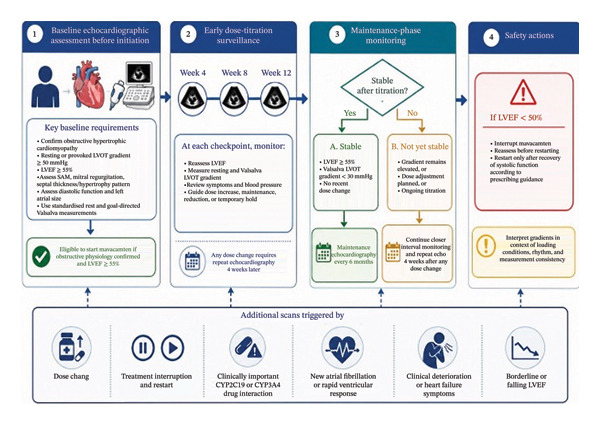
Echocardiography‐driven workflow for mavacamten therapy in obstructive hypertrophic cardiomyopathy. The figure summarises baseline echocardiographic confirmation of obstructive physiology and LVEF ≥ 55%, early dose‐titration surveillance, maintenance‐phase monitoring using LVEF and Valsalva LVOT gradient thresholds and additional scans triggered by dose changes, treatment interruptions, drug interactions, atrial fibrillation or clinical deterioration.

Through structured protocols and critical interpretation, echocardiography enables personalised and guideline‐aligned use of mavacamten. As cardiac myosin inhibitor therapy expands, echo laboratories will need robust workflows that integrate regulatory thresholds, exercise testing when indicated, diastolic and strain assessment and careful management of atrial fibrillation and drug interactions (see Figure 1).

## Author Contributions

Ali Nizam, Ibrahim Antoun, Liz Hodson, G. André Ng, and Harshil Dhutia: conceptualisation, methodology, validation, and writing of the original manuscript. Ali Nizam, Ibrahim Antoun, Liz Hodson, G. André Ng, and Harshil Dhutia contributed to critical review and editing.

## Funding

No funding was received for this research.

## Disclosure

All authors have approved the final manuscript.

## Consent

The authors have nothing to report.

## Conflicts of Interest

The authors declare no conflicts of interest.

## Data Availability

The study did not generate new data.
